# Analysis of SARS-CoV-2 Transmission by Airborne Droplets in a Restaurant Outbreak: A CFD Approach

**DOI:** 10.1155/cjid/5892658

**Published:** 2025-05-19

**Authors:** Yuezhu Chen, Xiaoman Jiang, Yong Yue

**Affiliations:** ^1^Chengdu Center for Disease Control and Prevention (Chengdu Institute of Health Supervision), Chengdu, Sichuan, China; ^2^Center for Disease Control and Prevention of Chengdu Hi-Tech Industrial Development Zone, Chengdu, Sichuan, China

**Keywords:** airborne transmission, CFD simulation, coughing, droplet, SARS-CoV-2

## Abstract

Restaurants have played a vital role in spreading the respiratory virus due to the invalidation of certain preventive behaviors such as mask wearing. We analyzed a coronavirus disease 2019 (COVID-19) outbreak involving two clusters in a restaurant to analyze SARS-CoV-2 transmission by airborne droplets, including aerosols, in a restaurant outbreak. Computational fluid dynamics (CFD) was used to simulate the spread of respiratory droplets generated by coughing. The cough jet was modeled as a turbulent jet to study the dispersion of expiratory droplets, with the realizable k-ε model being applied in this simulation. This outbreak involved six diners (A, B, D, E, F, and G) in two clusters (X and Y). But the two clusters were seated at two tables separated by over 3 m from each other, while none of the 18 patrons at the other seven tables, even patrons at neighboring tables, became infected. Upon further investigation, we found that the index case in Cluster X coughed violently with his head facing posterior to the right when Diner F entered the restaurant and passed the posterior side of the index case. Adequate droplets were ejected from the index case and were inhaled by Diner F or trapped by the surfaces of Diner F's hands, clothing, and belongings. The virus-laden droplets and aerosols generated by coughing can be responsible for inhalation or contamination of surfaces that they fall onto, leading to spread of the disease.

## 1. Introduction

The COVID-19 outbreak was an unprecedented global crisis with severe crowding in medical resources and international economic recession [1–3]. The transmission route of SARS-CoV-2 is certainly worthy of further in-depth study aimed at fighting the potential resurgence of coronavirus diseases [4–6]. It is widely known that the SARS-CoV-2 infection relies on the spreading of virus-containing respiratory droplets generated from expiratory events such as coughing or sneezing or even simply talking or breathing [7]. But the limitations of earlier understandings of droplet transmission alone were manifested during the COVID-19 pandemic. The transmission of SARS-CoV-2 by aerosols generated from expiratory events above, including their generation, movement, inhalation, and deposition, also needs to be taken into account [8]. How far droplets and aerosols can move is related to how far droplet-borne diseases can transmit [9]. And the mean residence time of SARS-CoV-2–laden droplets and aerosols was demonstrated to be related to release height, air exchange rate, relative humidity (RH), ambient temperature, and viral load [10].

Mask-wearing was a practical solution during the COVID-19 pandemic. However, masks need to be removed when dining out. Thus, restaurants have played a vital role in spreading the SARS-CoV-2 virus. Restaurants had opted or been forced to close completely in many countries and regions during the peak periods of the pandemic because there are no mask proof during mealtime and possibly high occupancy and mobility [11, 12]. For many emerging international tourist cities, the catering industry is an ever-growing industry which can receive numerous domestic and international guests. In face of the large population, high density and human mobility of mega city especially, epidemic prevention measures of restaurants are indeed crucial. To reduce the risk of transmission, there are a lot of precautions such as equipping with partitions in the restaurant, using serving chopsticks, and measuring temperature when walking into a restaurant [13]. In view of the important place of restaurants in the transmission of SARS-CoV-2 virus, the policies associated with restaurant management need to be optimized.

The diameter of droplets generated from speaking or coughing range from 1 μm to 1000 μm [14, 15]. Droplets greater than 5 μm in diameter are commonly assumed as respiratory droplets while droplets less than 5 μm in diameter as aerosols. Large virus-laden droplets can travel rather limited distance and contaminate surfaces, whereas small ones can persist a few minutes in the air and be inhaled before reaching the ground because of air resistance and evaporation. Therefore, analysis of airborne droplet transmission in a restaurant needs a whole picture of generated droplets in a full-size range and their motion trajectories.

To visualize the droplet spreading, spray droplet measurement and computational fluid dynamics (CFD) simulations are often used. CFD simulations are, relatively, more economical and more appropriate for retrospective studies. Some studies have simulated the SARS-CoV-2 transmission in indoor environments by CFD approach. For example, Li et al. simulated the spread of generated droplets and compared the virus-laden aerosol tracer with the specific location in the restaurant and found that inadequate ventilation played a role in the transmission of virus [16]. Wang et al. studied the transmission of SARS-CoV-2 in airliner cabins by simulating the dispersion of droplets generated by different strength of exhalations and found that most of the inhaled virus copies were laden by droplet nuclei which are less than 10 μm in diameter [17]. Jayaweera et al. discussed infection propensities and aerodynamic performance of SARS-CoV-2–loaded droplets and aerosols in varying confined spaces, including airplane, passenger car, and healthcare facilities [18]. Their research studies mainly focused on transmission in poorly ventilated or enclosed environments. Aerosols can usually persist a longer time and travel a longer distance in such environments, leading to a higher probability of infection.

In our study, the outbreak occurred in a well-ventilated, nonconfined space, which varied from the cases above. And only the cluster seated 3 m away with a partition divided in between was infected, which was anomalous. We studied the diffusion and distribution of SARS-CoV-2–laden droplets and aerosols in the well-ventilated restaurant by the CFD simulation to clarify the transmission and the corresponding weak link. Analysis of such unusual case can help us to identify omissions in disease control and prevention. Our study could be a scientific basis of supplement to policies on prevention and control of SARS-CoV-2 and even respiratory viruses.

## 2. Results

### 2.1. The Outbreak

**Figure 1:**
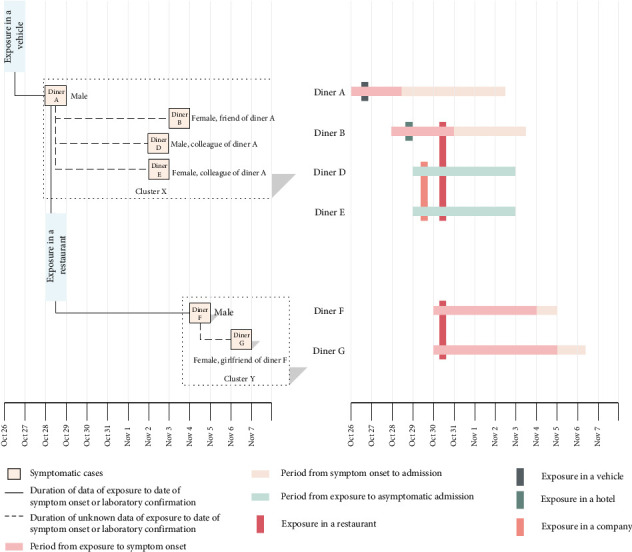
Cases in Clusters X and Y of COVID-19 in the restaurant and related timing of events.

Here, we report a detailed epidemiological study of an outbreak of a restaurant. For this study, we obtained a full video recording of the restaurant as a part of the epidemic investigation. All diners were monitored by a high-resolution surveillance camera at the time of exposure. The in-field measurements of the restaurant space were 10.89 m in length, 6.15-m wide, and 3.50-m high with a 2.70-m wide doorway. There were no solid doors or walls in the entrance of the space, and the wide-open entrance allowed air circulation of indoor and outdoor. The indoor air conditions on the day of the outbreak consisted of a temperature of 17°C, a RH level of 95%, and a wind speed of less than 0.5 m/s. Two unrelated clusters (X and Y) are associated with the COVID-19 outbreak in this restaurant. Cluster X (*n* = 5) consisted of the index case (Diner A), two local residents (Diners B and C) with no other history of travel or exposure, and two out-of-town residents (Diners D and E) who had just arrived in the local city. Cluster Y (*n* = 2) comprised a couple (Diners F and G) who are also local residents with no other history of travel or exposure. The two clusters dined at the same restaurant with a distance apart. Apart from Clusters X and Y, there were 18 diners staying in the same dining hall during the same time period. However, only Diners B, D, E, F, and G ended up with infection.  Figure 1 exhibits the cases in Clusters X and Y of COVID-19 in the restaurant and related timing of events.

### 2.2. A Doubtful Point

**Figure 2:**
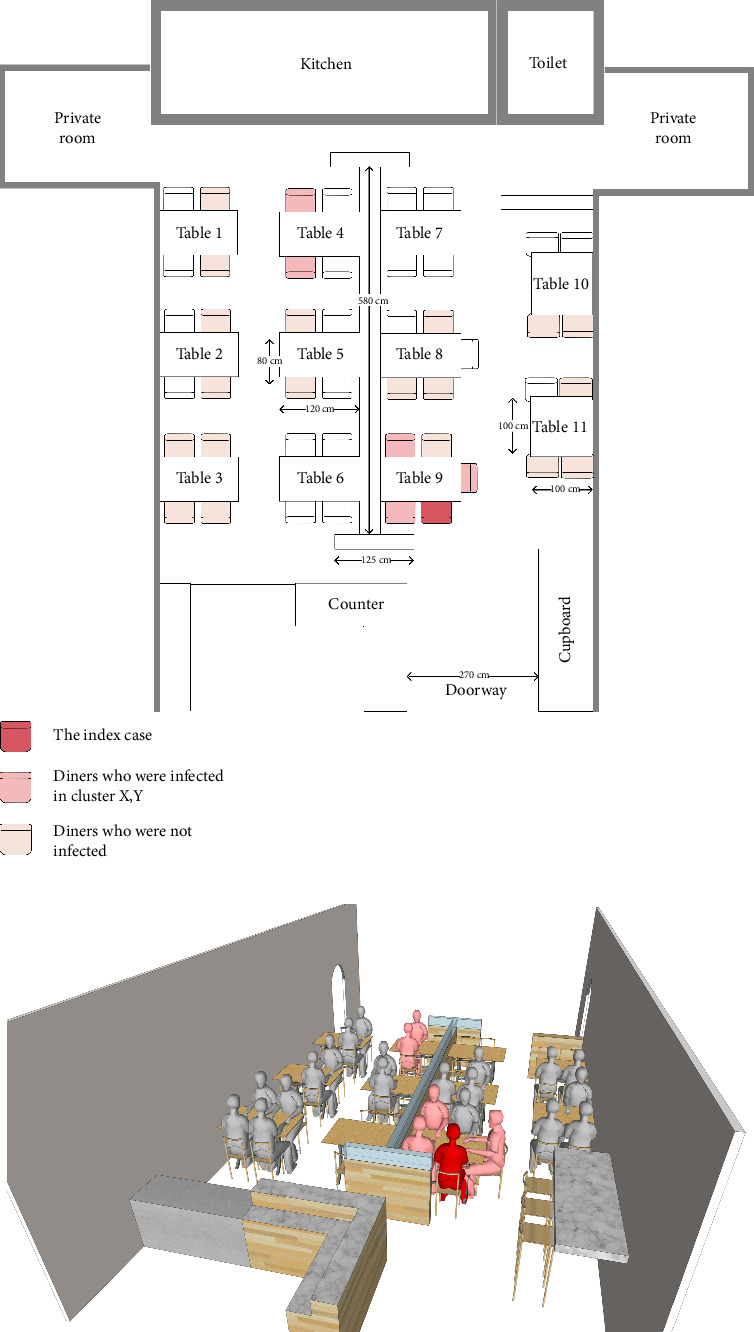
Diner positions and arrangement of the restaurant. (a) The top view; (b) the 3D view of the surveillance camera. Dark red represents the index case (Diner A), and light red represents the diners who were infected in Clusters X and Y (Diner B, D, E, F, and G).

Through a thorough field epidemiological investigation, we learned that Clusters X and Y dined at the same restaurant on the afternoon of 30th October. By examining video data related to the restaurant, we knew that Cluster X walked in the restaurant at 14 : 27, took their seats, stayed for about an hour, and left the restaurant at 15 : 22. Cluster Y entered the restaurant at 15 : 02, remained seated for about 40 min, and left the restaurant at 15 : 39. The time intervals of dining time of Clusters X and Y overlapped by 20 min. And both Clusters X and Y did not walk back and forth or use the toilet in the restaurant. Notably, though there was a long partition in the middle line of the restaurant, the two tables were positioned diagonally on the either side of the partition with a straight-line distance of over 3 m.  Figure 2 shows the diner positions and arrangement of the restaurant. Given the distance between the two clusters, the possibility of direct transmission during mealtime was relatively low. Quite apart from above, there were diners who ate at neighboring tables but were uninfected ultimately. Judging by the evidence above, how Cluster Y was infected with SARS-CoV-2 remained a doubtful point.

### 2.3. A Breakthrough in Investigation

**Figure 3:**
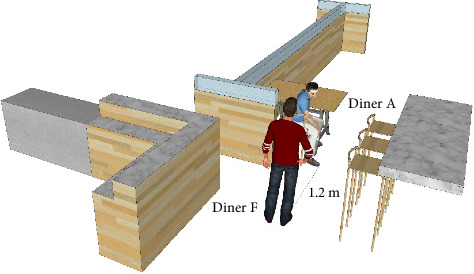
The relative position of Diner A and Diner F when Diner A walked into the restaurant. The shortest distance between them was 1.2 m.

On the way of looking for the association point of transmission between Clusters X and Y carefully, we found a special incident at 15 : 02. The index case (Diner A) coughed violently with his head facing posterior to the right when Diner F entered the restaurant and passed by the index case, as shown in Figure 3. After Diner F took his seat, he touched his belongings and clothing and then wore disposable plastic gloves with unwashed hands. Because of the specificity of the representative dishes in this restaurant, Diner F ate with his hands. In consideration of the fact that Diner F just passed by the index case who were violently coughing minutes ago, there is a very good chance that virus-containing droplets generated by coughing contaminated the surface of his skin, belongings, and clothing. Also, the virus-laden respiration droplets of aerosols could be inhaled by Diner F when he passed by the index case. The infection could be attributed to the direct inhalation or surface contamination or their synergism. After taking his seat, Diner F probably contaminated his hands by touching contaminated surfaces. The disposable plastic gloves were contaminated by unwashed hands. And the direct contact of contaminated gloves with the mouth may lead to Diner F's infection. Alternatively, the later direct contact of contaminated hands with the mucous membranes of the nose or eyes after leaving the restaurant can also cause infection.

### 2.4. Simulation of SARS-CoV-2 Transmission in the Restaurant—A CFD Approach

**Figure 4:**
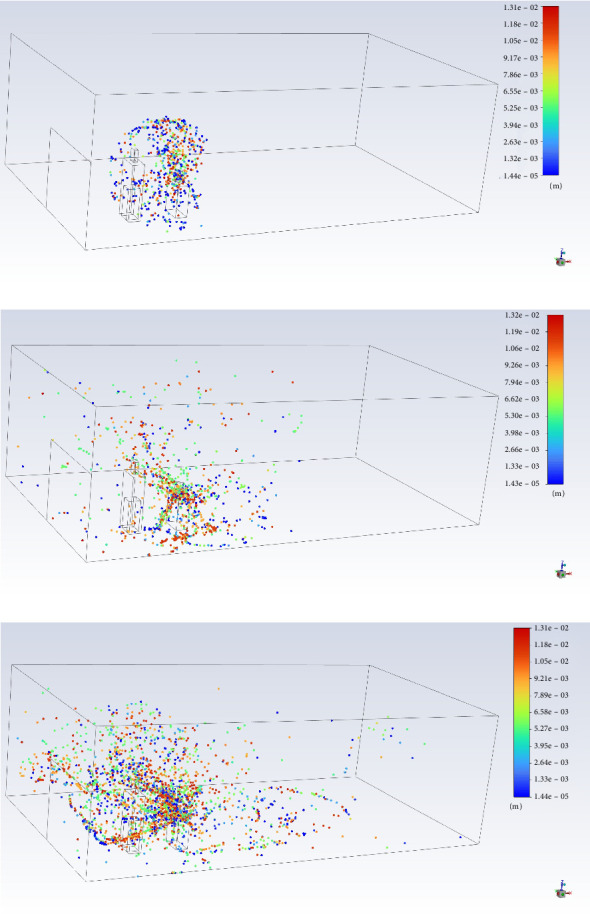
Temporal distributions of droplets generated by coughing at (a) 1 s, (b) 2 s, and (c) 3 s. Droplet traces colored by droplet diameter (m).

To illustrate how the infectious droplets generated by violent coughing spread in the restaurant, temporal distributions of droplets during the period when Diner F passed the posterior side of Diner A are displayed in Figure 4. Diner F was placed at the position nearest to Diner A. We can judge from the simulation that Diner F was in the coverage area of the cough cloud at the nearest position. There could be ample droplets ejected from Diner A and inhaled by Diner F or trapped by the surfaces of Diner F's hands, clothing, and belongings. The CFD simulation helped to establish a chain of evidence for Diner F's infection.

## 3. Methods

### 3.1. Basic Backgrounds and Settings for Simulation

A CFD approach was used to simulate the process of droplet spreading. The index case (Diner A) coughed at 15 : 02 with a sitting height of 112 cm. The violent cough lasted 7 s toward the 45-degree rear right of Diner A. During the first 3 s of the cough, Diner F walked into the restaurant and passed the posterior side of Diner A. The height of Diner A was 170 cm, and the shortest distance between Diners A and F was 120 cm. Diner F arrived at the nearest position at the third second of the cough. The geometric model size was established as the actual size of the restaurant.

### 3.2. Model and Boundary Conditions

We modeled the cough physically as a turbulent jet to study the airflow and the dispersion of expiratory droplets. The standard k-ε model, the RNG k-ε model, and the realizable k-ε model are usually used for turbulent flows. k-ε turbulence models consist of two transport equations to solve two transported variables which are the turbulence kinetic energy and the rate of dissipation. The realizable k-ε model was applied in this simulation for its good exactitude and convergence in indoor flow simulations [19]. As a turbulence model, the motion of the fluid flow generated by coughing was governed by Navier–Stokes equations.

In order to simulate the dispersion of respiratory droplets in this case using CFD, airflow velocity and flow rate are essential parameters for boundary conditions [20–22]. According to Kwon et al., the average initial coughing velocity measured using Particle Image Velocimetry was 15.3 m/s for males [21]. Chao et al. found from measurements that the average velocity of exhaled airflow was 11.7 m/s for coughing [14]. In some other studies, the velocity of respiratory exhalation flows was often set at 10 m/s for coughing [9, 20]. In our case, the initial velocity of the cough jet in the simulation was set at 10 m/s to avoid overimputation. According to Gupta et al., the cough peak flow rate of males ranged from 3 to 8.5 L/s [23]. Since Diner A coughed violently, the total flow rate was set at 6 L/s in the simulation. The mouth opening area was set to 4 cm^2^ as a constant since it is the average value for male subjects [23]. The diameter of droplets generated by coughing was set between 1 and 1000 μm to get a whole picture of the spread of droplets and droplet nuclei considering both aerosols and large droplets.

## 4. Discussion

The indoor–outdoor temperature difference and spatial connection can facilitate air convection, resulting in promoting ventilation. In this study, we assumed the restaurant to be a well-ventilated environment mainly based on the fact that its indoor space communicated with the outdoor space through the open doorway. According to Somsen et al., the number of droplets with an average diameter of 5 μm had halved within 30 s in a good ventilation room while those took about 1.4 min in a poor ventilation room [24]. Poor ventilation could definitely contribute to the persistence of aerosols and increase the infection risk [15]. In our study, infection occurred despite the good ventilation, which reminded us about the other focus of prevention and control.

According to the simulation results of the droplet spreading, infectious droplets and aerosols generated by coughing could be inhaled directly by Diner F or attached to the surfaces of Diner F's hands, clothing, and belongings. A high RH level could reduce the droplet inhalation because high RH enhances the condensation effect, helps growing droplet sizes, and reaches the ground before traveling a long horizontal distance [9, 25]. Considering a comprehensive condition of a 95% RH level and a well-ventilated indoor environment, the long-range transmission possibility was not completely ruled out but slim, which could also be supported by the fact that none of the other patrons in the restaurant were infected.

The results of this study should be considered in light of the following limitations. The evaporation and motion of droplets can be influenced by environmental factors such as air speed and RH besides the sizes of droplets [26, 27]. In our simulation model, the effects of those conditions are considered to be negligible because of the relatively high RH level and the relatively low wind speed. However, low RH leads to the rapid evaporation of the liquid phase and size reduction of droplets, increasing the time droplets and droplet nuclei suspended in air. The influence of wind speed on droplet movements is complicated and highly relied on the steadiness of the wind [25]. When it comes to an indoor environment with a low RH level and high wind speed, evaporation of droplets in respiratory jets and flow pattern of wind should be taken into account. In the simulation, the turbulent jet was considered to be originated from a single cough. But in real cases, the coughing process is closer to a sequential cough in which the flow rate may not be set as a constant and vary over time.

We can extend findings of our research by studying the numerical assessment of simulation results in indoor spaces. Although the persistence of the activity of SARS-CoV-2 particles in those airborne droplets appears to be sufficient to pose a threat [28], the question that how many SARS-CoV-2 particles in the virus-laden droplets may be enough to initiate an infection needs further study. Based on the viral load in the droplets and aerosols, the inspiratory tidal volume and respiratory rate of susceptible individuals, the critical number of viral particles required to infect a single individual, the size and volume of droplets emitted through the infected individual's expiratory activities, as well as the area and layout of the indoor environment, a function can be established to describe the relationship between infection probability and the duration of stay.

When we peered inside the case to get a whole picture of the transmission, the evidence of field epidemiology and simulation prompted us to link the infection to a special incident occurred within a few seconds. In order to block the transmission of respiratory viruses in similar cases, preventing the diffusion of droplets and aerosols in the cough jet generated by virus carriers and improving hygiene habits of susceptible individuals are two key aspects. There are two critical links that deserve our attention in this transmission route. First, Diner A did not cover his mouth and nose when he coughed violently. Even though he could not wear a mask while having meals, he still could cover his mouth and nose with tissue or elbow to block the spreading of droplets in larger areas. Aerosols generated by coughing or sneezing can be inhaled and result in infection. Both small and large droplets can be responsible for contaminating nearby surfaces of another person's face, hands, or clothes that they fall onto, leading to spread of the disease by later contact with an uninfected person [29]. Second, Diner F did not wash his hands before eating with them. If he washed his hands or used disinfection wipes right after getting seated, the risk of being infected could be reduced correspondingly. When dining out, the habit of hand washing before meals and covering the mouth and nose while coughing or sneezing could help reduce the risk of infection. This demonstrated that individual-level measures such as cough etiquette and hand washing are important especially in restaurants. Our study suggests that the government should reinforce publicity and education to improve the civil awareness of personal hygiene, with restaurants providing wipes for hand disinfection in parallel.

From another aspect, thanks to the well-ventilated environment and the partition in the crowded dining room, only Clusters X and Y of all the diners ended up with infection in our case. This indicated that some of the epidemic preventive policies were previously set in place and were of proven efficacy.

Most of the public health measures in restaurants could have been underestimated and somehow been neglected at first glance. How to put implementation into practice is what we want to emphasize here. As customers, we can pay more attention to personal hygiene. For restaurants, correct use of air conditioning or air supply system, improving toilet hygiene, and providing adequate hand rubs and hand disinfectants are good measures to practice. The government can provide antiepidemic materials and publicity materials to support restaurants if necessary. While such actions may seem unremarkable, they are proven to be useful to reduce the risk of infection. We can start with small movements and build a safe dining environment for the whole public and the city.

## Data Availability

The data that support the findings of this study are available on request from the corresponding author. The data are not publicly available due to privacy or ethical restrictions.
